# Noninvasive Focused Ultrasound Stimulation Can Modulate Phase-Amplitude Coupling between Neuronal Oscillations in the Rat Hippocampus

**DOI:** 10.3389/fnins.2016.00348

**Published:** 2016-07-22

**Authors:** Yi Yuan, Jiaqing Yan, Zhitao Ma, Xiaoli Li

**Affiliations:** ^1^Institute of Electrical Engineering, Yanshan UniversityQinhuangdao, China; ^2^School of Electrical and Control Engineering, North China University of TechnologyBeijing, China; ^3^State Key Laboratory of Cognitive Neuroscience and Learning and IDG/McGovern Institute for Brain Research, Beijing Normal UniversityBeijing, China; ^4^Center for Collaboration and Innovation in Brain and Learning Sciences, Beijing Normal UniversityBeijing, China

**Keywords:** focused ultrasound stimulation, neuronal oscillation, phase-amplitude coupling, hippocampus, ultrasonic intensity

## Abstract

Noninvasive focused ultrasound stimulation (FUS) can be used to modulate neural activity with high spatial resolution. Phase-amplitude coupling (PAC) between neuronal oscillations is tightly associated with cognitive processes, including learning, attention, and memory. In this study, we investigated the effect of FUS on PAC between neuronal oscillations and established the relationship between the PAC index and ultrasonic intensity. The rat hippocampus was stimulated using focused ultrasound at different spatial-average pulse-average ultrasonic intensities (3.9, 9.6, and 19.2 W/cm^2^). The local field potentials (LFPs) in the rat hippocampus were recorded before and after FUS. Then, we analyzed PAC between neuronal oscillations using a PAC calculation algorithm. Our results showed that FUS significantly modulated PAC between the theta (4–8 Hz) and gamma (30–80 Hz) bands and between the alpha (9–13 Hz) and ripple (81–200 Hz) bands in the rat hippocampus, and PAC increased with incremental increases in ultrasonic intensity.

## Introduction

Neuronal oscillations provide a mechanism for forming cell assemblies and coordinating cell assemblies by linking the activity of multiple neurons (Wiley, 2010). Neuronal oscillations, which are the complex cognitive functions of the brain that occur from the synergistic interaction of multiple neurons, are mainly involved in cognitive processes, including feature binding, selective attention, and memory. In neuronal oscillations, the amplitude of a faster rhythm is coupled to the phase of a slower rhythm; this is termed phase-amplitude coupling (PAC) and reflects the interactions between local micro-scale and systems-level macro-scale neuronal ensembles. Therefore, PAC can be used as an index of cortical excitability and network interactions (Klausberger et al., 2003; Knight, 2007; Haider and McCormick, 2009; Voytek et al., 2010). Recent findings have shown that PAC was a mechanism for working memory capacity and the discrete nature of perception. PAC also plays an important role during sleep (Penny et al., 2008; Wang et al., 2014). In brief, PAC is a new index that reflects the dynamic interactions between neuronal oscillations.

Noninvasive focused ultrasound stimulation (FUS) of isolated turtle nerve fibers was first performed in 1929 (Newton, 1929). A previous study showed that the mechanism of FUS involves ultrasound-induced cavitation of nanometric bilayer sonophores, which can induce a complex mechanoelectrical interplay that leads to excitation, primarily through the effect of currents induced by membrane capacitance changes of neurons (Plaksin et al., 2014). Changes in neural networks induced by FUS may be caused by changes in the firing rhythm of several neurons. Low-intensity ultrasound can directly modulate neuronal activity in peripheral nerves (Mihran et al., 1990; Tyler, 2011), elicit action potentials in hippocampal slices (Tyler et al., 2008), synchronous oscillations in the intact hippocampus (Tufail et al., 2010) and stimulate the retina (Menz et al., 2013). Furthermore, it can noninvasively stimulate the intact mouse motor cortex (Tufail et al., 2010). Recently, low-intensity focused ultrasound was used to modulate visuomotor behavior in monkeys (Deffieux et al., 2013). Focused ultrasound was also applied to modulate the activity of the primary somatosensory cortex in humans (Legon et al., 2014). Tufail and Yoo et al used pulses of low-frequency (250–700 kHz) ultrasound to modulate brain function (Tufail et al., 2010; Yoo et al., 2011). Mehiæ et al. used a higher frequency (2 MHz), pulsed and focused ultrasound to stimulate the brain of lightly anesthetized mice (Mehiæ et al., 2014). Compared with transcranial direct current stimulation (tDCS) and repetitive transcranial magnetic stimulation (rTMS), FUS has a higher spatial resolution (< 2 mm; Tufail et al., 2010; Bystritsky et al., 2011; Yoo et al., 2011). To date, there have been no reports on the effect of low-intensity ultrasound on PAC between neuronal oscillations. In this study, we focused exclusively on the effect of FUS on PAC between neuronal oscillations derived from the hippocampus. A thorough study of the relationship between PAC and ultrasonic intensity is necessary to evaluate the effects of FUS on neuromodulation and to provide a reference for choosing ultrasonic intensities when applying FUS. Our study investigated PAC between neuronal oscillations in the rat hippocampus induced by different ultrasonic intensities of FUS. Local field potentials (LFPs) were recorded from the rat hippocampus before and after FUS. The effects of FUS on PAC between the theta (4–8 Hz) and gamma (30–80 Hz) bands and between the alpha (9–13 Hz) and ripple (81–200 Hz) bands were analyzed using the phase-amplitude coupling index (PACI).

## Materials and methods

### Experimental setup for FUS and data acquisition

**Figure 1:**
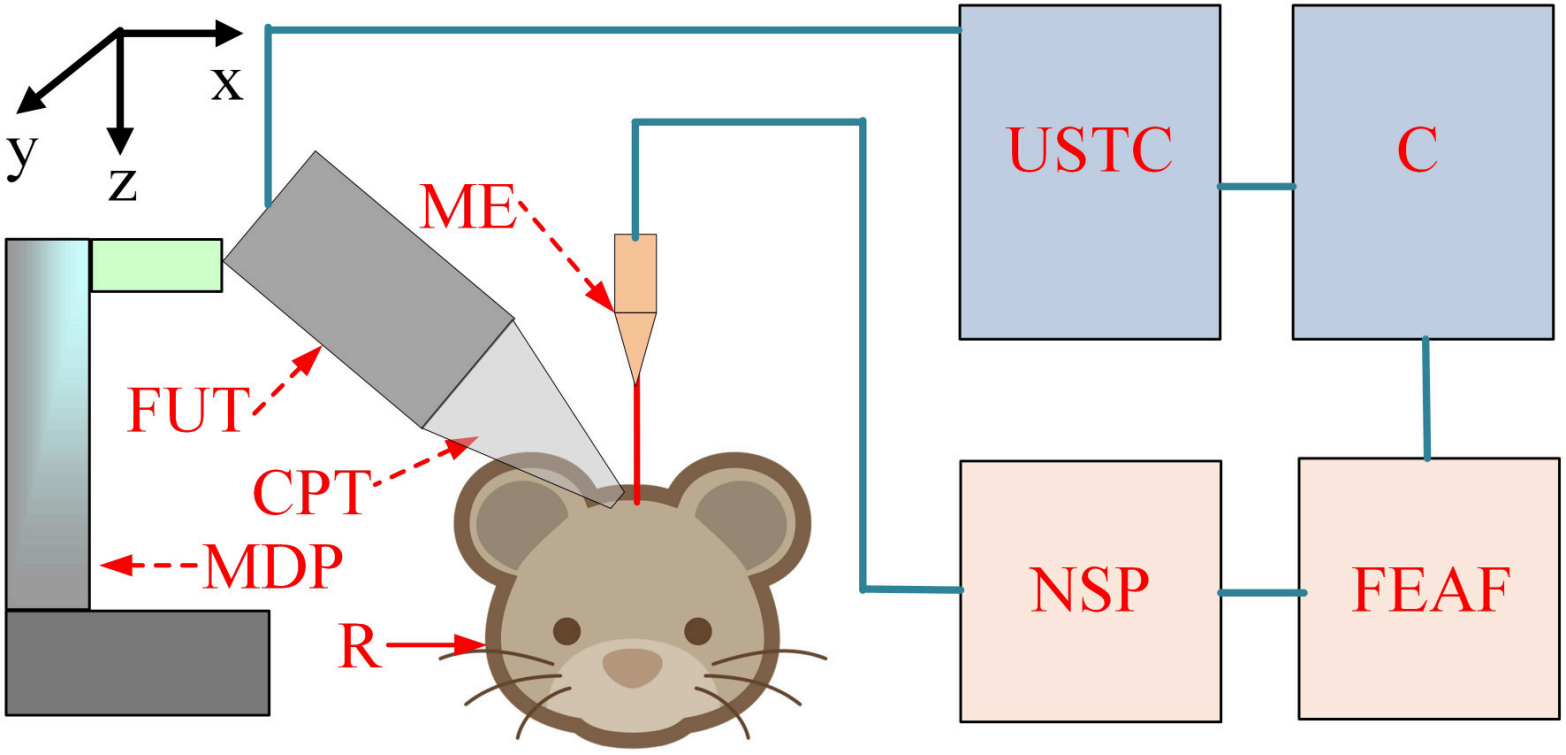
**The schematic of the experiment setup**. C, computer; USTC, ultrasonic transmitter card; ME, microelectrode; FUT, focused ultrasound transducer; CPT, conic plastic tube; R, rat; MDP, manual displacement platform; NSP, neural signal processor; FEAF, front-end amplifier.

The schematic of the experimental setup is shown in Figure 1. The pulse signals were generated by an ultrasonic transmitter and receiver card (USB-UT350T, Ultratek, USA) that drove the focused ultrasound transducer. In our study, the ultrasound transducer, with a bandwidth of 50% and focal length of 30 mm, was driven at a high frequency (2.5 MHz). The active volume of the ultrasound probe was ~625.36 mm^3^ and the diameter of the ultrasound intensity focus field was ~4.5 mm. A single stimulation consisted of 4 cycle (1.6 μs) ultrasound pulses with a pulse repetition frequency of 500 Hz and a duration of 160 ms (80 repetitions). An ultrasonic sound power measuring instrument with a diameter of 9.5 mm was used to measure the average intensity of the ultrasound (YP0511F, Hangzhou, China). The spatial-average pulse-average intensities of the ultrasound were 3.9, 9.6, and 19.2 W/cm^2^.

LFP signals from the hippocampus were recorded using a 16-channel microelectrode (GBMA-S16, Blackrock Microsystems, USA) and amplified using a 128-channel front-end amplifier (Cerebus, 128 channels, Blackrock Microsystems, USA). The analog signals were converted into digital signals using a 128-channel neural signal processor (Cerebus128 channels, Cyberknetics, USA), which was then transmitted to a computer for data storage and processing. The data were digitized at a sample rate of 30 kHz, and a low-pass filter with a 250 Hz cutoff frequency for the LFPs was set in the Cerebus system. A heating blanket was used to maintain normal body temperature in the rats. A cold-light source and microscope were used for surgery and a shielding net was used to prevent outside electrical interference.

### Animal surgery and anesthesia

A total of six Sprague-Dawley rats (3-month-old males, body weights ~270 g) were used in the experiment. All procedures were carried out in accordance with the Animal Ethics and Administrative Council of Yanshan University and Hebei Province, China. Surgical anesthesia was induced with sodium pentobarbital (3%, 5 mg/100 g, i.p.). The anesthetized rats were fixed on the stereotaxic apparatus (ST-5ND-C, Stoelting Co., USA) with ear bars and a clamping device. The fur covering the rat's skull was shaved, and the skin was cleaned with a 0.9% sodium chloride physiological solution. The scalp was cut along the midline of the skull, and the subcutaneous tissue and periosteum were removed. The location of the hippocampus was determined using an atlas. A section of the skull was removed to expose the brain tissue in an area of ~0.5 × 0.5 cm. After completion of the surgical procedure, a 16-channel metal microelectrode (GBMA-S16, Blackrock Microsystems, USA) was inserted into the hippocampus. The anteroposterior (AP), mediolateral (ML), and dorsoventral (DL) coordinates of the center of the recording electrode were -5.3, 3.4, and 3 mm, respectively.

### Experimental procedure

In the ultrasound stimulation experiment, the anesthetized rats were fixed on the stereotaxic apparatus (ST-5ND-C, Stoelting Co., USA) with ear bars and a clamping device. The focused transducer was aimed at the rat hippocampus by adjusting a three-axis manual displacement platform (Zolix, China). A 27 mm plastic cone was filled with degassed ultrasound gel and used to couple the ultrasound transducer to the cortex over the hippocampus. The ultrasound transducer then transmitted an ultrasonic wave. The focused ultrasonic wave passed through the ultrasonic coupling medium and stimulated the brain tissue to induce noninvasive brain neuromodulation. The angle between the ultrasound and the recording microelectrode was ~60⋅.

### PAC analyses

**Figure 2:**
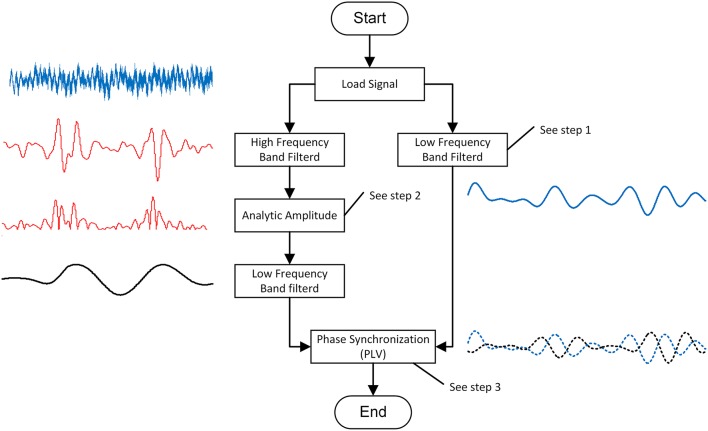
**The schematic of PAC calculation algorithm**.

PAC is the coupling degree index between the low-frequency phase and the high-frequency amplitude. In this study, we modified Voytek's method to calculate the PACI between the low and high frequencies (Voytek et al., 2013). The process (shown in Figure 2) of this calculation method included three steps.

Step 1: band-pass filter

A harmonic wavelet was used to provide an unbiased and consistent estimation of the EEG power spectrum (Kapiris et al., 2005). The harmonic wavelet was chosen instead of the more commonly used Morlet wavelet because it is orthogonal and its expression is simple. The wavelet transform passes a filter ψ(⦁)over a time series *x*(*t*) to obtain a finite number of filtered signals.

(1) $$PACI=\left|\frac{1}{K}\sum_{k=1}^{K-1}\exp\left(i\left(\phi_{l}\left[k\right]-\phi_{h}\left[k\right]\right)\right)\right|,$$

where ψ(⦁) is the basic or mother wavelet function and *a* and τ denote the scale factor and the translation of the origin, respectively. The variable 1/*a* gives the frequency scale, and τ gives the temporal location of an event. *W*_*x*_(*a*, τ) can be interpreted as the “energy” of *x* of scale *a* at *t* = τ. Moreover, the harmonic wavelet function is defined as

(2) $$\begin{array}{r} \psi_{m,n}\left(t\right)=\frac{e^{jn2\pi t}-e^{jm2\pi t}}{j\left(n-m\right)2\pi t}, \end{array}$$

where *m* and *n* are the real scale parameters but not necessarily integers. For the discrete time series *t* = τ, the wavelet transform is expressed as

(3) $$\begin{array}{r} W_{S}\left(n\right)=\overset{N-1}{\underset{n=0}{\sum }}x_{n}\psi_{0}^{*}\left(\frac{n-N}{S}\right), \end{array}$$

where ^*^ indicates the complex conjugate. By varying the wavelet scale *s* and translating along the localized time index *k*, one can construct a picture that shows both the amplitude of any feature vs. the scale and how this amplitude varies with time. The band pass filtered discrete sequence *x*_*n*_ is defined as

(4) $$\begin{array}{r} x_{filt}\left(f\right)=2\times real\left(W\left(f\right)\right). \end{array}$$

Step 2: analytic amplitude

For the filtered signal *x*_*filt*_*s*__, we used the absolute value to reflect the power of the signal. The power of the discrete sequence*x*_*filt*_*s*__ is defined as

(5) $$\begin{array}{r} P\left(s\right)=|x_{{filt}_{s}}|, \end{array}$$

where |⦁| indicates the absolute value.

Step 3: phase synchronization

Phase synchronization describes the phase relationship of the two signals. In this study, we applied the Hilbert transform to estimate the phases (ϕ_l_, ϕ_h_) for the two signals: (i) the low-frequency oscillation and (ii) the low-frequency band-pass filtered high–frequency oscillation amplitude.

Then, the PACI between the two signals was defined by the following equation

(6) $$W_{x}\left(a,\tau \right)=\frac{1}{\sqrt{\left|a\right|}}\int x\left(t\right)\psi \left(\frac{t-\tau }{a}\right)dt,$$

where *PACI* is the phase-locking value between the ongoing phase ϕ_*l*_ and ϕ_*k*_, and *k* is the time index. PAC differences between the control and FUS at the different ultrasonic intensities were analyzed for all subjects using Kruskal-Wallis with Tukey-Kramer *post-hoc* test that is a non-parametric statistical test method.

## Results

**Figure 3:**
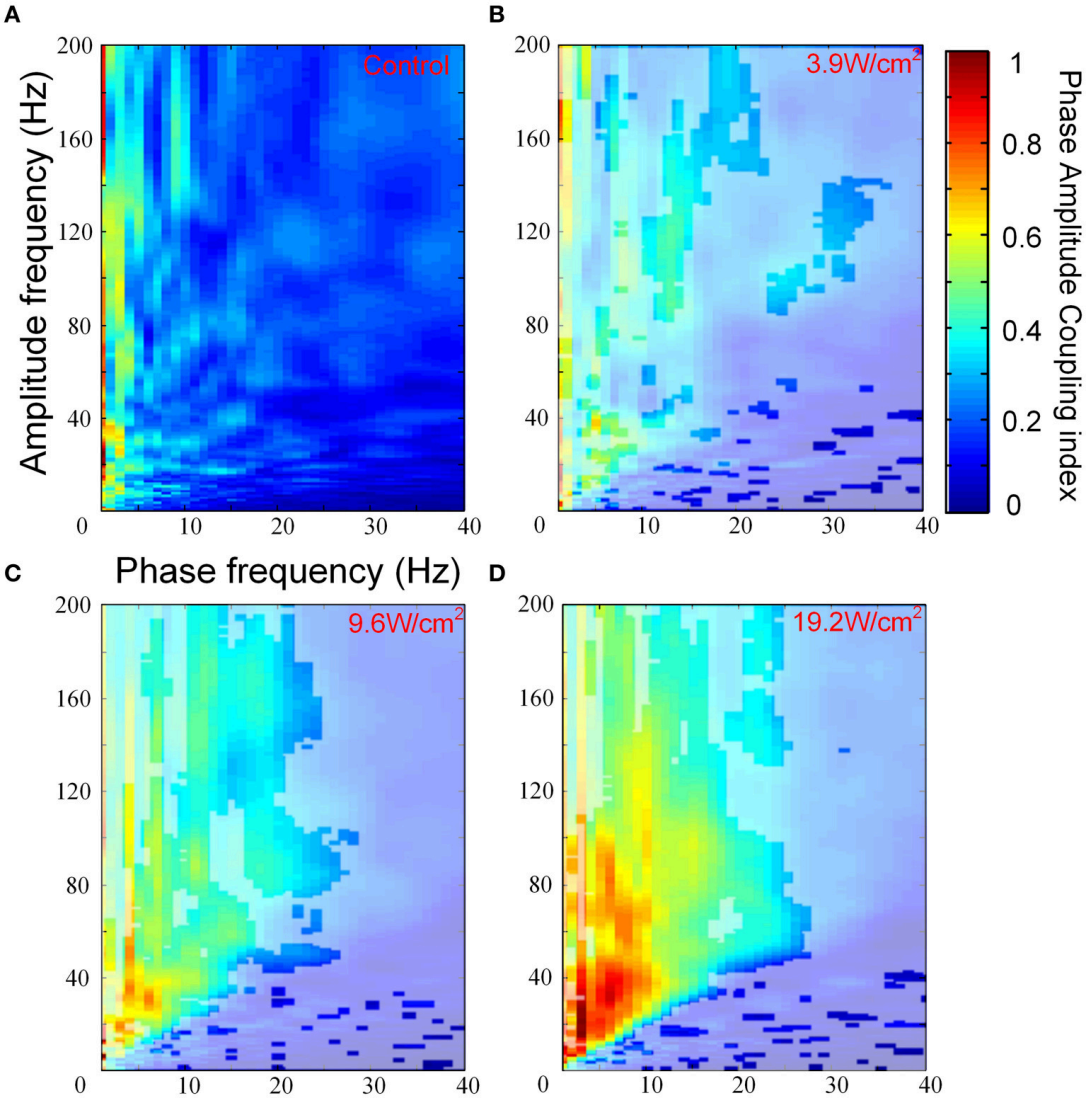
**(A)** The images of the PACI as a function of the analytic phase (1–40 Hz) and the analytic amplitude (1–200 Hz) for the control (*n* = 6). **(B–D)** The images of the PACI as a function of the analytic phase (1–40 Hz) and the analytic amplitude (1–200 Hz) for the FUS with different ultrasonic intensities: **(B)** 3.9 W/cm^2^, **(C)** 9.6 W/cm^2^, **(D)** 19.2 W/cm^2^ (*n* = 6).

We investigated PAC that was induced by FUS at different ultrasonic intensities. Figures 3A–D show the images of the PACI as a function of the analytic phase (1–40 Hz) and analytic amplitude (1–200 Hz) for the control and FUS at the different intensities. In Figures 3B–D, the area not covered by white shadows indicated that they are mean ranks significantly different from the control group (Kruskal-Wallis with Tukey-Kramer *post-hoc* test). When the ultrasonic intensity was 3.9 W/cm^2^, the PACI showed that there was a significant difference between FUS and the control in a small frequency range (Figures 3A,B). These changes were mainly reflected in the theta, alpha and gamma frequency bands. Compared to the ultrasonic intensity (3.9 W/cm^2^), the PACI in the large frequency range was significantly enhanced with an ultrasonic intensity of 9.6 W/cm^2^ (Figure 3C). This enhancement was obvious in the theta and gamma frequency bands, the alpha and gamma frequency bands, and the ripple frequency bands. When the ultrasonic intensity was 19.2 W/cm^2^ (Figure 3D), the PACI was further increased compared to the ultrasonic intensity of 9.6 W/cm^2^. However, it is worth noting that there were no significant changes in the frequency bands. Based the comparison of the PACI between the three ultrasonic intensities and the control, we found that the change in PAC was obvious in the theta and gamma frequency bands and the alpha and ripple frequency bands. Therefore, to further analyze the effects of ultrasonic intensity on the PACI, we separately calculated the mean PACI in the theta and gamma frequency bands and the alpha and ripple frequency bands.

**Figure 4:**
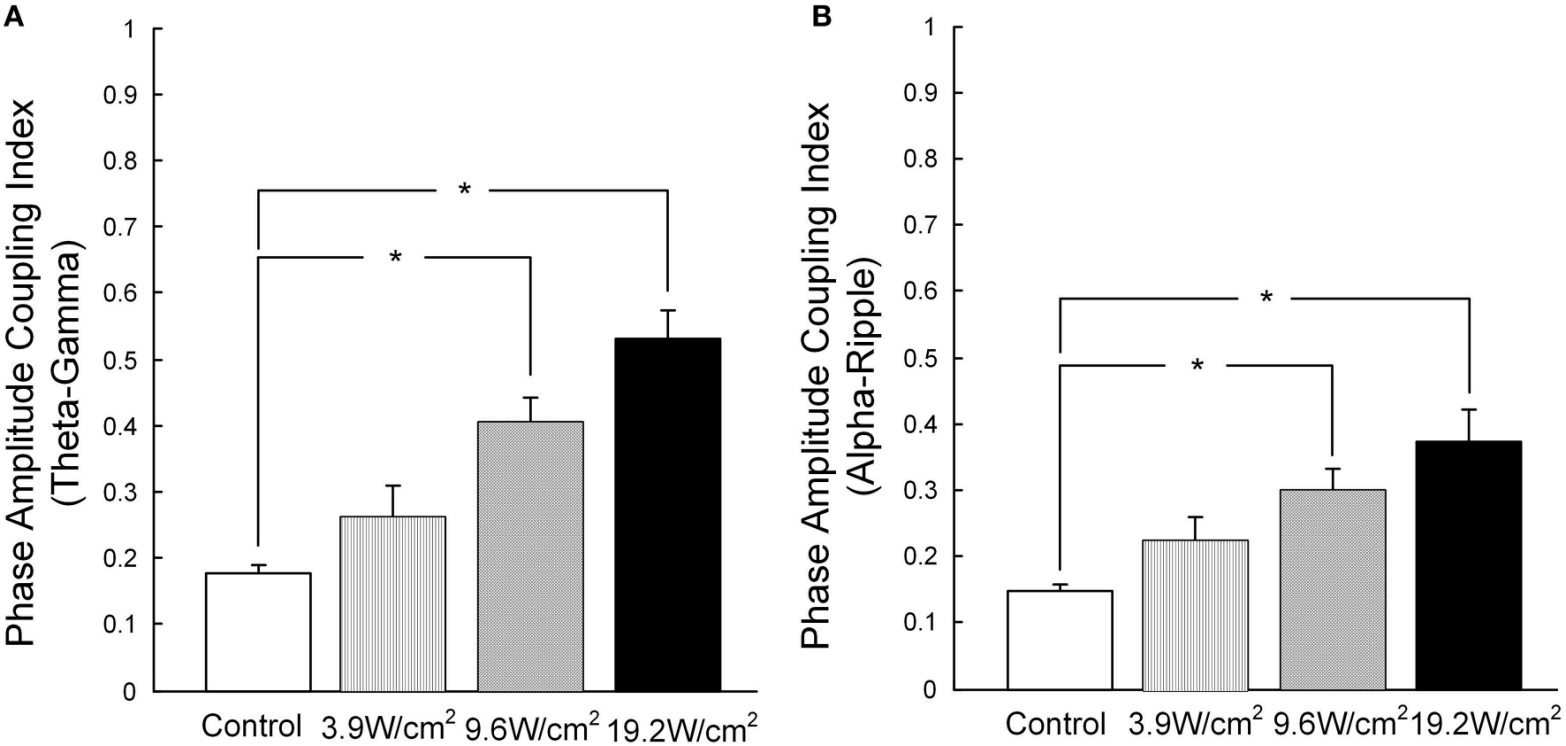
**(A)** The mean PACI of theta (4–8 Hz) and gamma coupling (30–80 Hz) in the control and with ultrasonic intensities of 3.9, 9.6, and 19.2 W/cm^2^ [mean ± SEM, *n* = 6, Chi-Sq (3, 20) = 16.49, ^*^p = 0.0009: mean ranks mean ranks significantly different, Kruskal-Wallis with Tukey-Kramer *post-hoc* test]. **(B)** The mean PACI of alpha (9–13 Hz) and ripple coupling (81–200 Hz) in the control and with ultrasonic intensities of 3.9, 9.6, 19.2 W/cm^2^ [mean ± SEM, *n* = 6, Chi-Sq (3, 20) = 14.25, ^*^p = 0.0026: mean ranks significantly different, Kruskal-Wallis with Tukey-Kramer *post-hoc* test].

Furthermore, in Figures 3A–D, we quantitatively computed the mean PACI, which is equal to the total PACI divided by the total points. As is shown in Figure 4A, the mean PACI between the theta (4–8 Hz) and gamma (30–80 Hz) bands for the control and the different ultrasonic intensities (3.9, 9.6, and 19.2 W/cm^2^) was 0.17 ± 0.01, 0.26 ± 0.04, 0.41 ± 0.03, and 0.53 ± 0.04, respectively [mean ± SEM, *n* = 6, Chi-Sq (3, 20) = 16.49, ^*^p = 0.0009: mean ranks significantly different, Kruskal-Wallis with Tukey-Kramer *post-hoc* test). Compared with the control, the mean PACI between the theta and gamma band increased by 1.53-, 2.41-, and 3.12-fold for the ultrasonic intensities of 3.9, 9.6, and 19.2 W/cm^2^, respectively. Therefore, the mean PACI between the theta and gamma band significantly increased as the ultrasonic intensity increased. A similar result was found for the alpha (9–13 Hz)-ripple (81–200 Hz) coupling (Figure 4B). The mean PACI between the alpha (9–13 Hz) and ripple (81–200 Hz) bands for the control and the different ultrasonic intensities (3.9, 9.6, and 19.2 W/cm^2^) was 0.15 ± 0.01, 0.22 ± 0.03, 0.30 ± 0.03, and 0.37 ± 0.05, respectively [mean ± SEM, *n* = 6, Chi-Sq (3, 20) = 14.25, ^*^p = 0.0026: mean ranks significantly different, Kruskal-Wallis with Tukey-Kramer *post-hoc* test]. Compared with the control, the mean PACI between the alpha and ripple band increased by 1.46−, 2.01−, and 2.46−fold for the ultrasonic intensities of 3.9, 9.6, and 19.2 W/cm^2^, respectively. These results show that the FUS can significantly enhance the PAC index between the theta and gamma band and between the alpha and ripple band as the ultrasonic intensity increases.

## Discussion

In our previous work (Yuan et al., 2015), we used focused ultrasound with different parameters to stimulate the rat hippocampus. We recorded LFPs evoked by FUS in the rat hippocampus. The mean absolute power of the LFPs was calculated using the Welch algorithm at the delta, theta, alpha, beta and gamma frequency bands. The experimental results demonstrate that the mean absolute power of the LFPs at the different frequency bands increases as the ultrasound power increases. However, we did not pay particular attention to the effect of the ultrasonic parameters on PAC of LFP signals. Subsequently, we found that ultrasonic power can influence PAC of the LFP between the low and high-frequency bands, specifically, in the theta and gamma frequency bands and the alpha and ripple frequency bands.

Previous studies have shown that PAC is associated with brain functions. For example, human memory strength can be predicted by theta-frequency phase-locking of singles (Rutishauser et al., 2010). Therefore, investigating the relationship between PAC and FUS is important. We calculated the mean PACI between the alpha and ripple band and between the theta and gamma band at different ultrasonic intensities (3.9, 9.6, and 19.2 W/cm^2^). Our results indicated that the PACI between the theta and gamma band and between the alpha and ripple band were closely connected with the ultrasonic intensity. We can alter the amplitude characteristics of the high-frequency LFPs by modulating the phase characteristics of the low-frequency LFPs by adjusting the ultrasonic intensity. Cognitive abilities (such as memory storage, retrieval, etc.) are closely related to PAC (Mann and Mody, 2010); therefore, we can modulate cognitive abilities by altering ultrasonic intensity. PAC can also optimize ultrasonic intensity by modulating brain oscillations during the application of FUS to treat neurological diseases.

In our study, we analyzed PAC in the rat hippocampus using three ultrasonic intensities (3.9, 9.6, and 19.2 W/cm^2^). To obtain a more accurate understanding of the functional relationship between PAC and different ultrasonic intensities, we plan to stimulate the rat hippocampus with additional ultrasound intensities in future studies. The range of acoustic parameters can affect neural activity. A previous study showed that the success of ultrasound stimulation increases as a function of both the acoustic intensity and acoustic duration (King et al., 2013). Perhaps other ultrasonic parameters, including the center frequency, stimulus frequency, duration and number of cycles, can also affect PAC in the hippocampus. In this study, we only quantitatively analyzed the effect of different ultrasonic intensities of FUS on PAC in the rat hippocampus. In future studies, we will evaluate the effect of the other ultrasonic parameters mentioned above on PAC in the hippocampus.

Because ultrasound at high intensities or during long exposures can burn and damage tissues, it is very important to control the ultrasonic intensity when FUS is applied to modulate brain activity. In this study, the maximum ultrasonic intensity was 19.2 W/cm^2^, which was not only below the maximum recommended limit for diagnostic imaging applications (190 W/cm^2^) but was also below the 23.87 W/cm^2^ that was used to modulate the activity in the primary somatosensory cortex in humans (Nyborg, 2001). Therefore, the ultrasonic dose in our experiment is safe. Studies have shown that neuromodulation using TMS is based on the motor threshold (Awiszus, 2003). However, TMS cannot modulate brain activity due to the weak intensity of the magnetic field. This study demonstrates that PAC between neuronal oscillations in the rat hippocampus can be altered using different ultrasonic intensities, which may support the use of PAC between neuronal oscillations to select the appropriate ultrasound intensity for FUS.

In summary, PAC between neuronal oscillations in the rat hippocampus can be altered by FUS, and the PACI increased as the ultrasonic intensity increased. To our knowledge, this is the first study of its kind to demonstrate the effect of FUS with different ultrasonic intensities on PAC between neuronal oscillations.

## Author contributions

YY and XL designed and coordinated the study, YY, JY, and ZM carried out experiment and data process, and drafted the manuscript. All authors gave final approval for publication.

### Conflict of interest statement

The authors declare that the research was conducted in the absence of any commercial or financial relationships that could be construed as a potential conflict of interest.
